# Supplementary material for: ALLO-1- and IKKE-1-dependent positive feedback mechanism promotes the initiation of paternal mitochondrial autophagy
Source: Nat Commun. 2024 Feb 17;15:1460. doi: 10.1038/s41467-024-45863-2 (PMC10874384; doi:10.1038/s41467-024-45863-2)
Supplement: Supplementary file 1 — Supplementary Information [file 41467_2024_45863_MOESM1_ESM.pdf]

Supplementary material for ALLO-1- and IKKE-1-dependent positive feedback mechanism promotes the initiation of paternal mitochondrial autophagy

Taeko Sasaki[1,2], Yasuharu Kushida[2], Takuya Norizuki[1], Hidetaka Kosako[3],
Ken Sato[2*], Miyuki Sato[1*]

[1]Laboratory of Molecular Membrane Biology, Institute for Molecular and Cellular Regulation, Gunma University, Maebashi, Gunma 371-8512, Japan

[2]Laboratory of Molecular Traffic, Institute for Molecular and Cellular Regulation, Gunma University, Maebashi, Gunma 371-8512, Japan

[3]Division of Cell Signaling, Fujii Memorial Institute of Medical Sciences, Institute of Advanced Medical Sciences, Tokushima University, Tokushima 770-8503, Japan

*Correspondence: m-sato@gunma-u.ac.jp; sato-ken@gunma-u.ac.jp

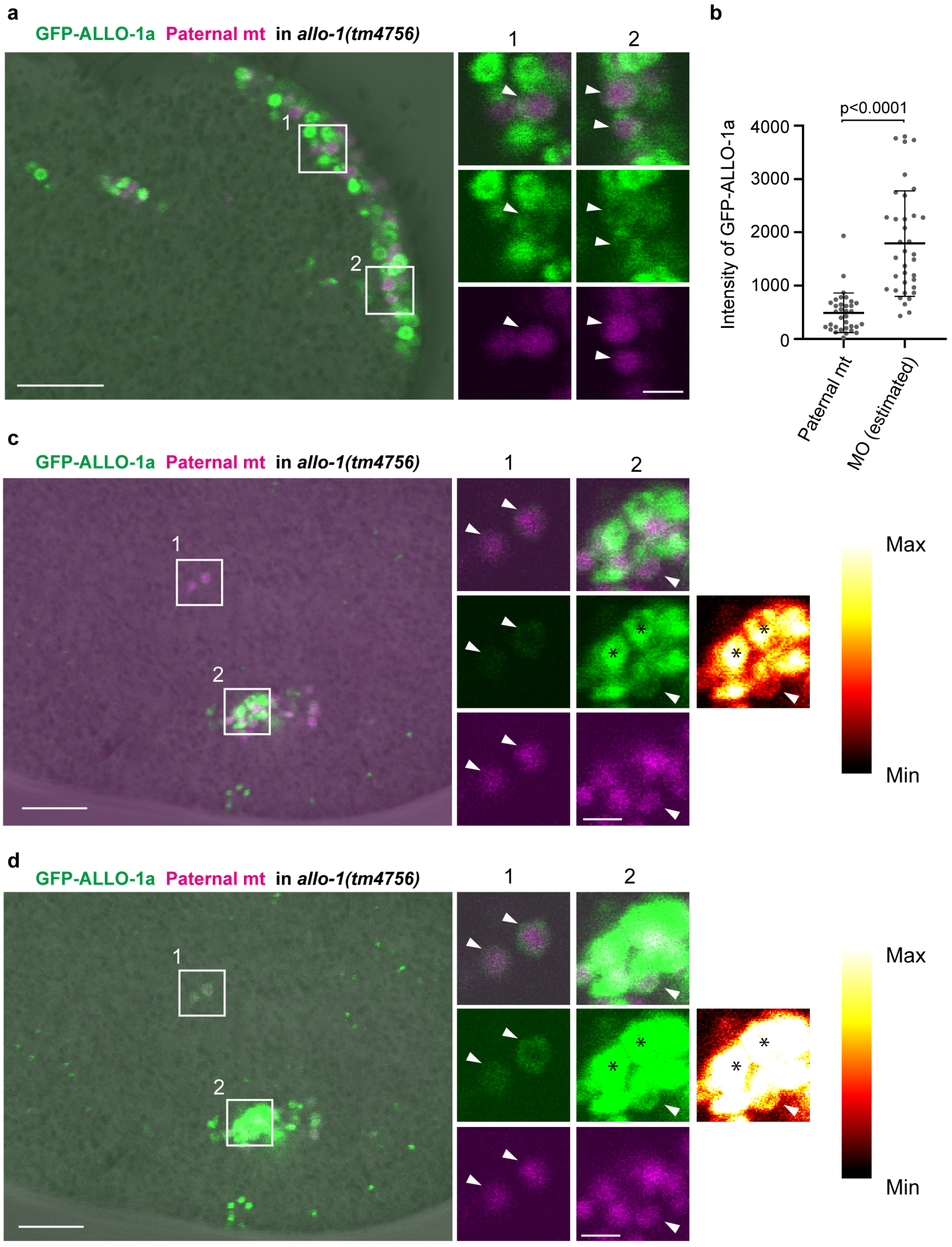

Supplementary Fig. 1 Localization patterns of GFP-ALLO-1a around paternal mitochondria
a) Image in Fig. 1c with adjusted brightness. Arrowheads indicate paternal mitochondria with GFP-ALLO-1a signals. **b)** Intensity of GFP-ALLO-1a around paternal organelles. Bright spots outside the paternal mitochondria were considered as membranous organelles (MOs) and analyzed. $n = 34$ (paternal mitochondria) or $n = 36$ (estimated MOs) from 6 zygotes in pseudo-cleavage stage. p value was calculated using the two-tailed Mann–Whitney U test. Error bar represents the mean ± standard deviation (SD). **c)** Another zygote (pseudo-cleavage stage) expressing GFP-ALLO-1a. GFP-ALLO-1a (green) weakly colocalized with paternal mitochondria (magenta; arrowheads). Asterisks indicate estimated MO signals. The heatmap image of GFP in enlarged panel 2 was shown on the rightmost panel. **d)** Zygote shown in c with adjusted brightness. Although the GFP signal of paternal mitochondria was visible, estimated MO signals (asterisks) were saturated. GFP-ALLO-1a was expressed in the *allo-1* mutant background. Scale bars, 5 µm for the left panel and 1 µm for the enlarged right panels.

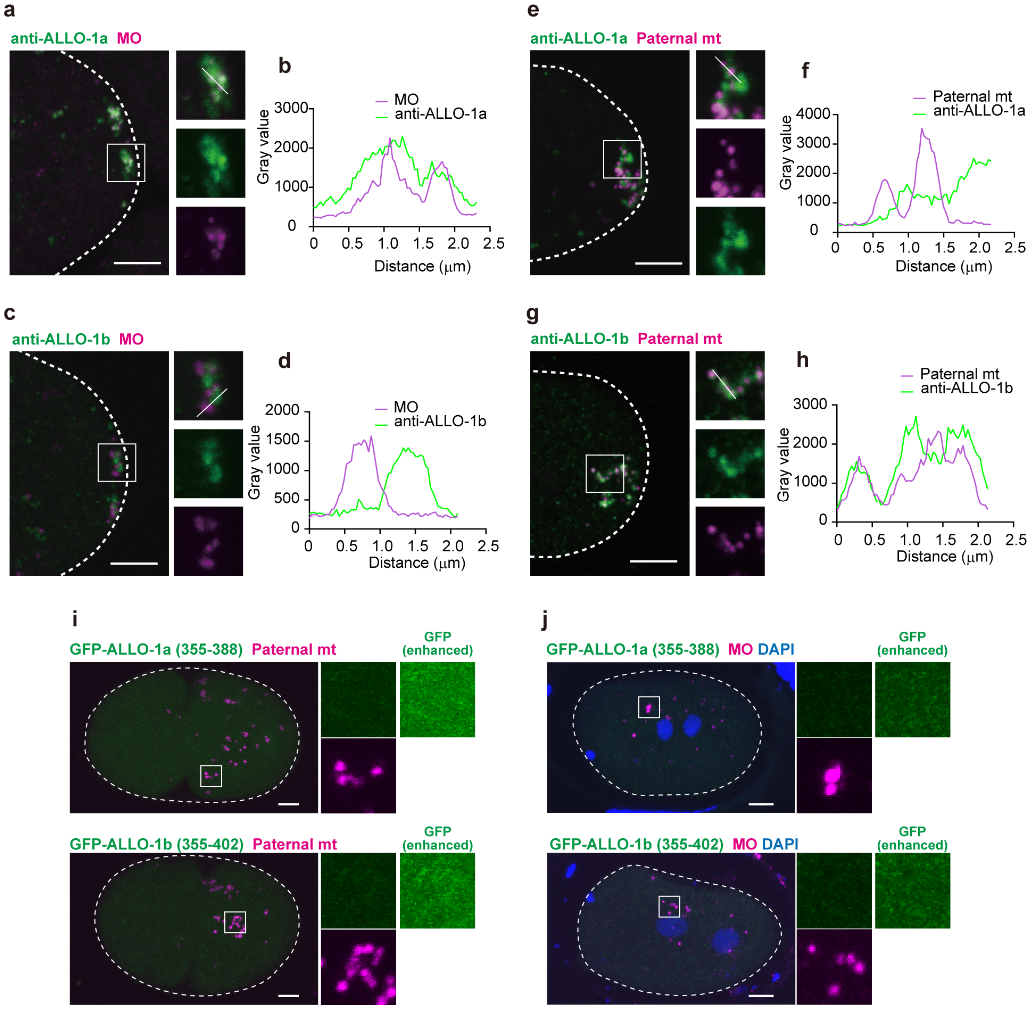

Supplementary Fig. 2 Localization patterns of ALLO-1a and ALLO-1b

a–d) Localization patterns of ALLO-1a and ALLO-1b around membranous organelles (MOs). The zygotes were dissected from wild type and stained with 1CB4 (MO, magenta) and anti-ALLO-1a (a, b) or anti-ALLO-1b (c, d)-specific antibodies (green). Plot profiles of white lines in the upper right panels of a and c are shown in b and d, respectively. White dotted lines indicate outline of the zygotes. **e–h)** Localization patterns of ALLO-1a and ALLO-1b around paternal mitochondria (HSP-6-GFP; magenta). The zygotes were stained with anti-ALLO-1a (e, f)- or anti-ALLO-1b (g, h)-specific antibodies (green). Plot profiles of white lines in the upper right panels of e and g are shown in f and h, respectively. Egg shells are indicated by dotted white lines. Zygotes at the pronuclear expansion stage were shown in a-h. **i, j)** Localization patterns of ALLO-1a- or ALLO-1b-specific region. i) Transgenic worms expressing GFP-ALLO-1a (355-388) or GFP-ALLO-1b (355-402) (green) were mated with *him-5(e1490)* males expressing HSP-6-mCherry (magenta) under the sperm-specific *spe-11* promoter, and F1 zygotes around the pseudo-cleavage stage were dissected and observed. j) Zygotes expressing GFP-ALLO-1a (355-388) or GFP-ALLO-1b (355-402) (green) around the pseudo-cleavage stage were stained with 1CB4 (MO, magenta), and 4′,6-diamidino-2-phenylindole (DAPI; blue). White dotted lines indicate outline of the zygotes. Scale bars, 5 μm.

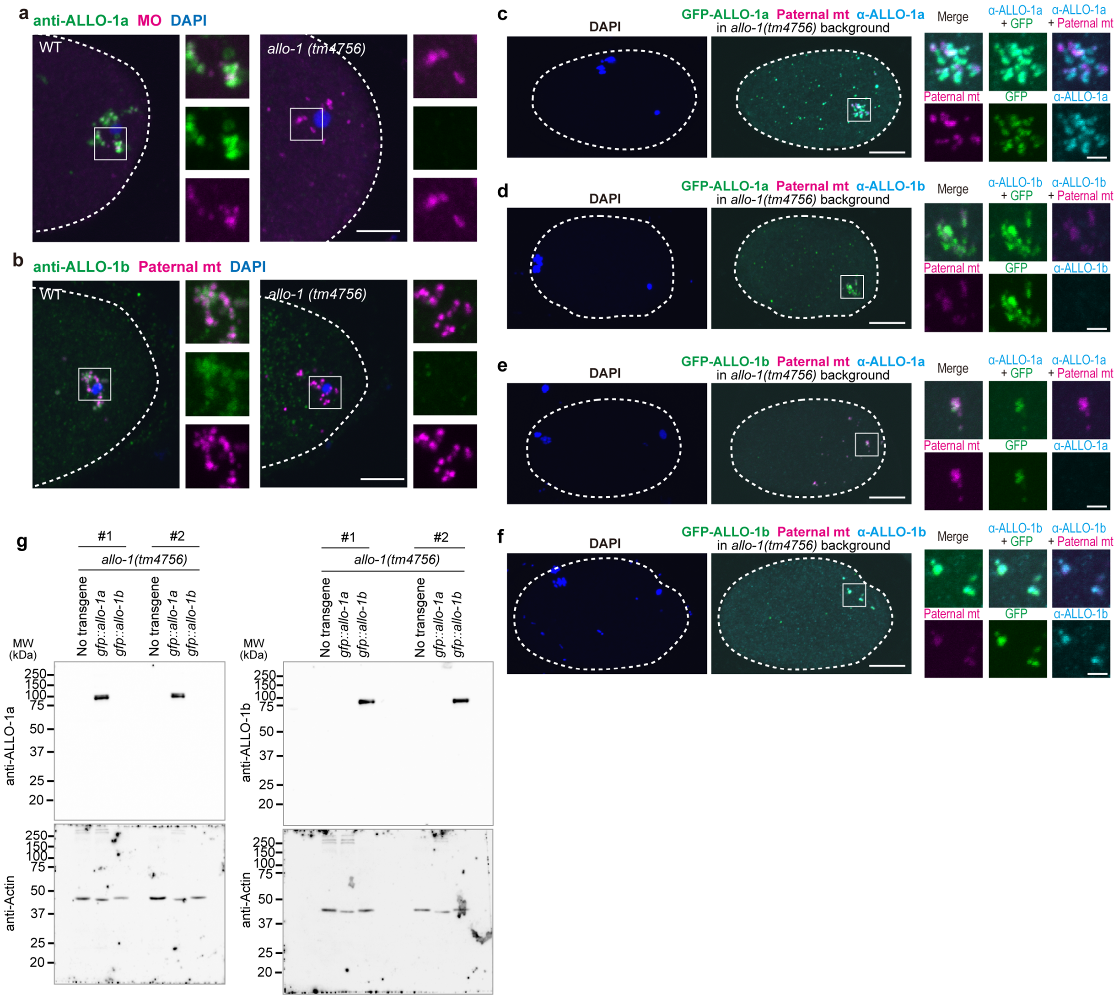

Supplementary Fig. 3 Confirmation of specificity of anti-ALLO-1a and anti-ALLO-1b antibodies

a) Specificity of anti-ALLO-1a antibody. The zygotes were dissected from wild type (WT) and *allo-1(tm4756)* and stained with a 1CB4 antibody, the marker of membranous organelles (MOs; magenta), anti-ALLO-1a antibody (green), and 4′,6-diamidino-2-phenylindole (DAPI; blue). **b)** Specificity of anti-ALLO-1b antibody. The zygotes were dissected from wild-type (WT) and *allo-1(tm4756)* hermaphrodites expressing HSP-6-GFP under the sperm-specific *spe-11* promoter (marker of paternal mitochondria; magenta) and stained with an anti-ALLO-1b-specific antibody (green) and DAPI (blue). **c, d)** Specificity of anti-ALLO-1a antibodies. The zygotes (meiosis II stage) were dissected from hermaphrodites expressing GFP-ALLO-1a (green) and sperm-specific HSP-6-mCherry (magenta) in the *allo-1(tm4756)* background and stained with an anti-ALLO-1a antibody or anti-ALLO-1b antibody (cyan), and DAPI (blue). **e, f)** Specificity of anti-ALLO-1b antibodies. The zygotes (meiosis II stage) were dissected from hermaphrodites expressing GFP-ALLO-1b (green) and sperm-specific HSP-6-mCherry (magenta) in the *allo-1(tm4756)* background and stained with an anti-ALLO-1a antibody or anti-ALLO-1b antibody (cyan), and DAPI (blue). Scale bars, 5 µm. **g)** Immunoblotting using anti-ALLO-1a and anti-ALLO-1b antibodies. Proteins were extracted from the fertilized eggs expressing GFP-ALLO-1a or GFP-ALLO-1b in the *allo-1(tm4756)* background and analyzed. The results of two experiments using independently obtained samples are shown (#1, #2).

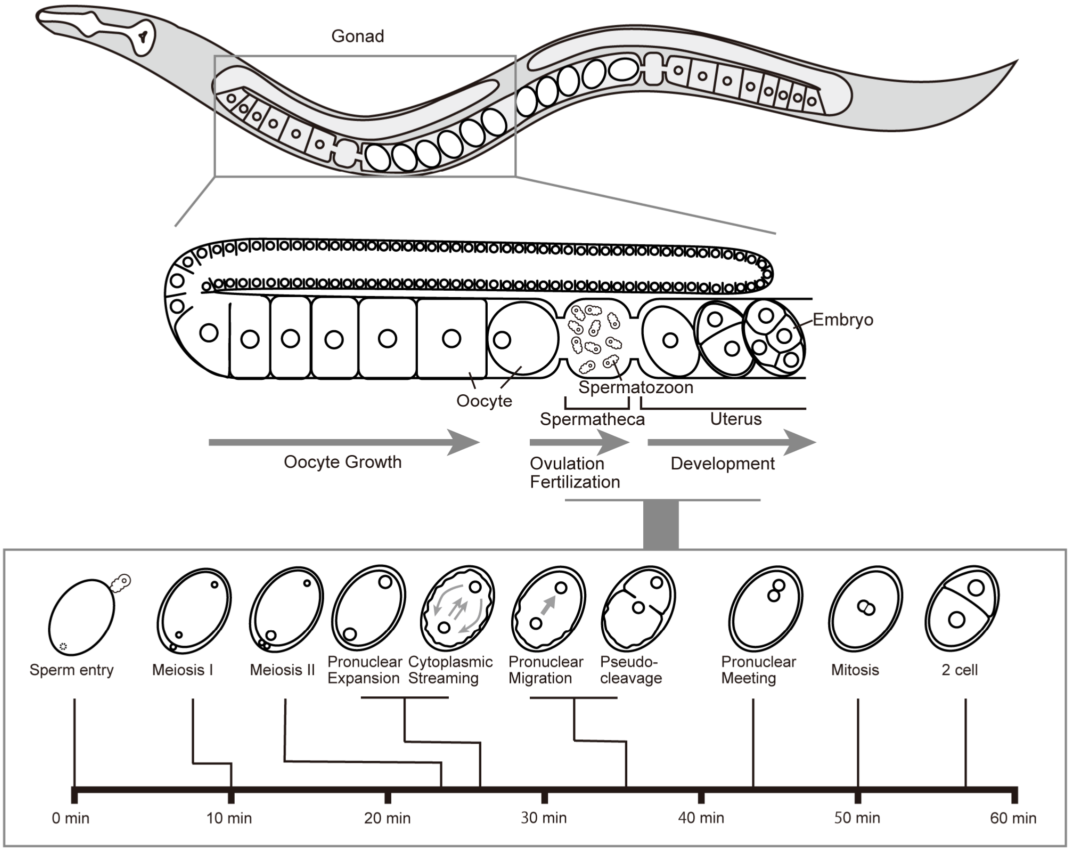

Supplementary Fig. 4. Schematic representation of *Caenorhabditis elegans* gonads

Caenorhabditis elegans hermaphrodites have two symmetrical pairs of gonads. Unfertilized eggs produced in the gonads are fertilized by passing through a spermatozoa-filled spermatheca and transferred to the uterus. The general time course of early embryogenesis at 20°C is shown in the bottom frame.[37] In most cases, the position of paternal organelles changed quickly because of ovulation and cytoplasmic streaming, which sometimes caused misalignment of the position of GFP and paternal mitochondria in the movies (Fig. 3; Supplementary Movie 1 and 2).

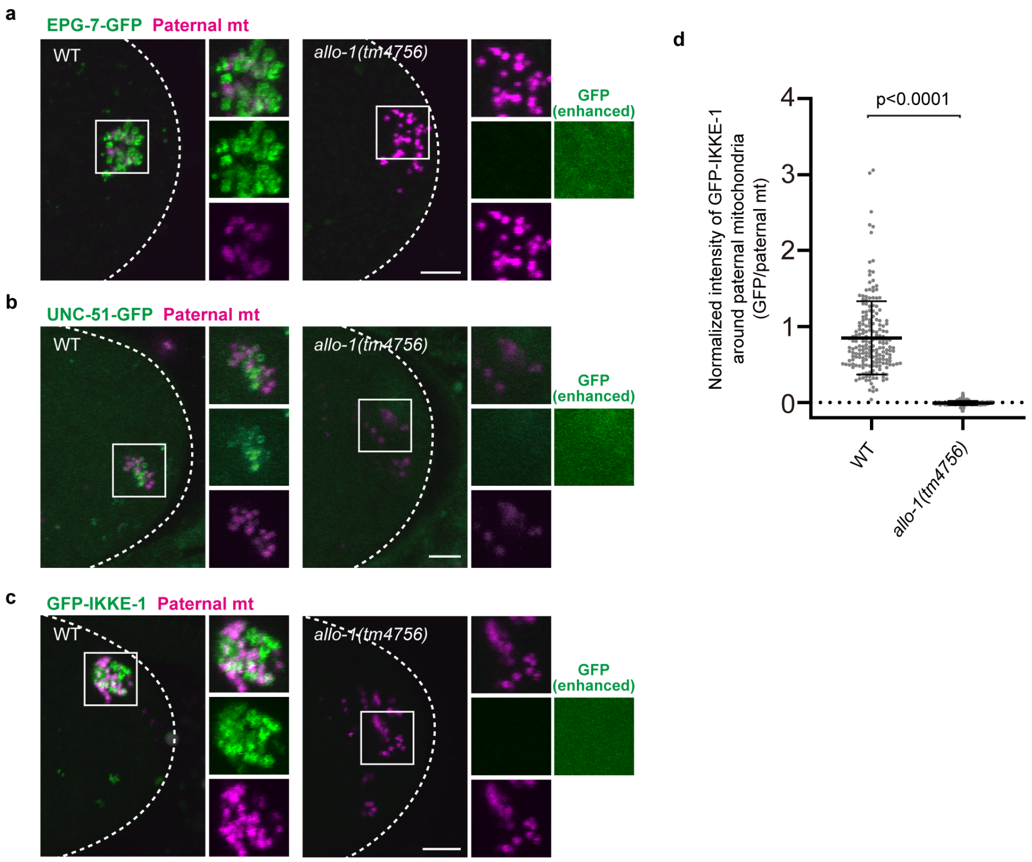

Supplementary Fig. 5. ALLO-1 regulates recruitment of the IKKE-1 and ULK complex during allophagy

a–c) Localization of EPG-7, UNC-51, and IKKE-1 around paternal organelles depends on ALLO-1 expression. EPG-7-GFP (a), UNC-51-GFP (b) or GFP-IKKE-1 (c) (green), and HSP-6-mCherry (paternal mitochondria; magenta) were observed in wild-type (WT) or *allo-1(tm4756)* mutant zygotes. Zygotes were observed around meiosis I for EPG-7-GFP and UNC-51-GFP, and around meiosis II for GFP-IKKE-1. Images with adjusted brightness of GFP in *allo-1(tm4756)* were shown in the upper right corner of each figure; no GFP localization was observed. Scale bars, 5 µm. In total, 10 (a), 4 (b), and 11 (c) zygotes were observed for each genotype, and similar patterns were observed in all zygotes. White dotted lines indicate outline of the zygotes. **d)** The intensity of GFP-IKKE-1. Intensity was quantified and normalized based on the fluorescence of paternal mitochondria. $n = 197$ (WT) and $n = 251$ (*allo-1(tm4756)*) paternal mitochondria or their clusters from 10 zygotes, respectively, were analyzed around meiosis II stage. p value was calculated using the two-tailed Mann–Whitney U test.

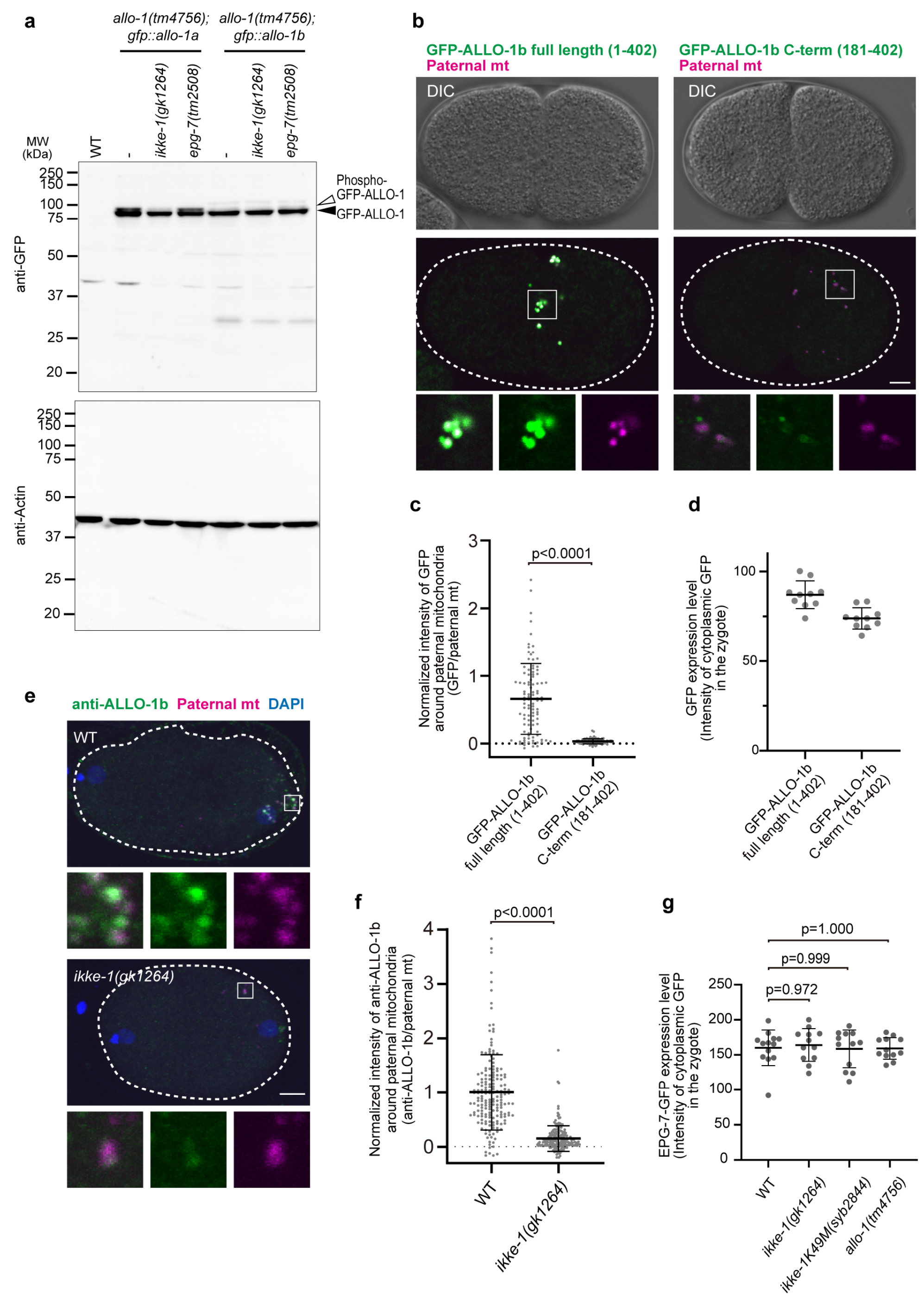

Supplementary Fig. 6. IKKE-1 is essential for ALLO-1b accumulation

a) Immunoblotting of GFP-ALLO-1a and GFP-ALLO-1b in the *epg-7* and *ikke-1* mutants. Whole-worm lysates were prepared from adult hermaphrodite of the *allo-1(tm4756)*, *allo-1(tm4756); ikke-1(gk1264)*, or *allo-1(tm4756); epg-7(tm2508)* mutant expressing GFP-ALLO-1a or GFP-ALLO-1b and analyzed. Whole-worm lysates of the wild type expressing no transgene (WT) was included as a control. **b, c)** N-terminal region is required for ALLO-1b accumulation. GFP-ALLO-1b or GFP-ALLO-1b C-term (green) and HSP-6-mCherry (paternal mitochondria; magenta) were observed in zygotes at the pseudo-cleavage stage. In c, the intensity of GFP was quantified and normalized based on the fluorescence of paternal mitochondria to correct for GFP attenuation because of the distance from the objective. *n* = 102 (GFP-ALLO-1b) and *n* = 142 (GFP-ALLO-1b C-term) paternal mitochondria or their clusters from 6 or 10

zygotes, respectively. Egg shells are indicated by white dotted lines. **d)** Expression level of GFP-ALLO-1b full and C-term. Intensity of cytoplasmic GFP was quantified outside the vicinity of the paternal organelles to compare GFP expression levels between transgenes. n = 10 zygotes for each genotype. **e, f)** Endogenous ALLO-1b failed to accumulate in the *ikke-1* deletion mutant. The zygotes were dissected from wild type (WT) or *ikke-1(gk1264)* expressing HSP-6-mCherry (paternal mitochondria; magenta) and stained with the anti-ALLO-1b (green) antibody and 4′,6-diamidino-2-phenylindole (DAPI; blue). Outline of the zygotes are indicated by white dotted lines. In f, the intensity derived from anti-ALLO-1b was quantified and normalized based on the fluorescence of paternal mitochondria. n = 180 (wild type) and n = 184 mitochondria or their clusters were analyzed from 10 zygotes. **g)** Expression level of EPG-7-GFP in *ikke-1* and *allo-1* mutants. Intensity of cytoplasmic GFP was quantified outside the vicinity of the paternal organelles to compare GFP expression levels between mutants. n = 13, 12, 12, or 12 zygotes for wild type, *ikke-1(gk1264), ikke-1(syb2844)*, or *allo-1(tm4756)*, respectively. p values were calculated using the two-tailed Mann–Whitney U test (c and f) or one-way ANOVA with TukeyHSD pairwise comparison test (g). Error bars represent the mean ± standard deviation (SD). Scale bars, 5 µm.

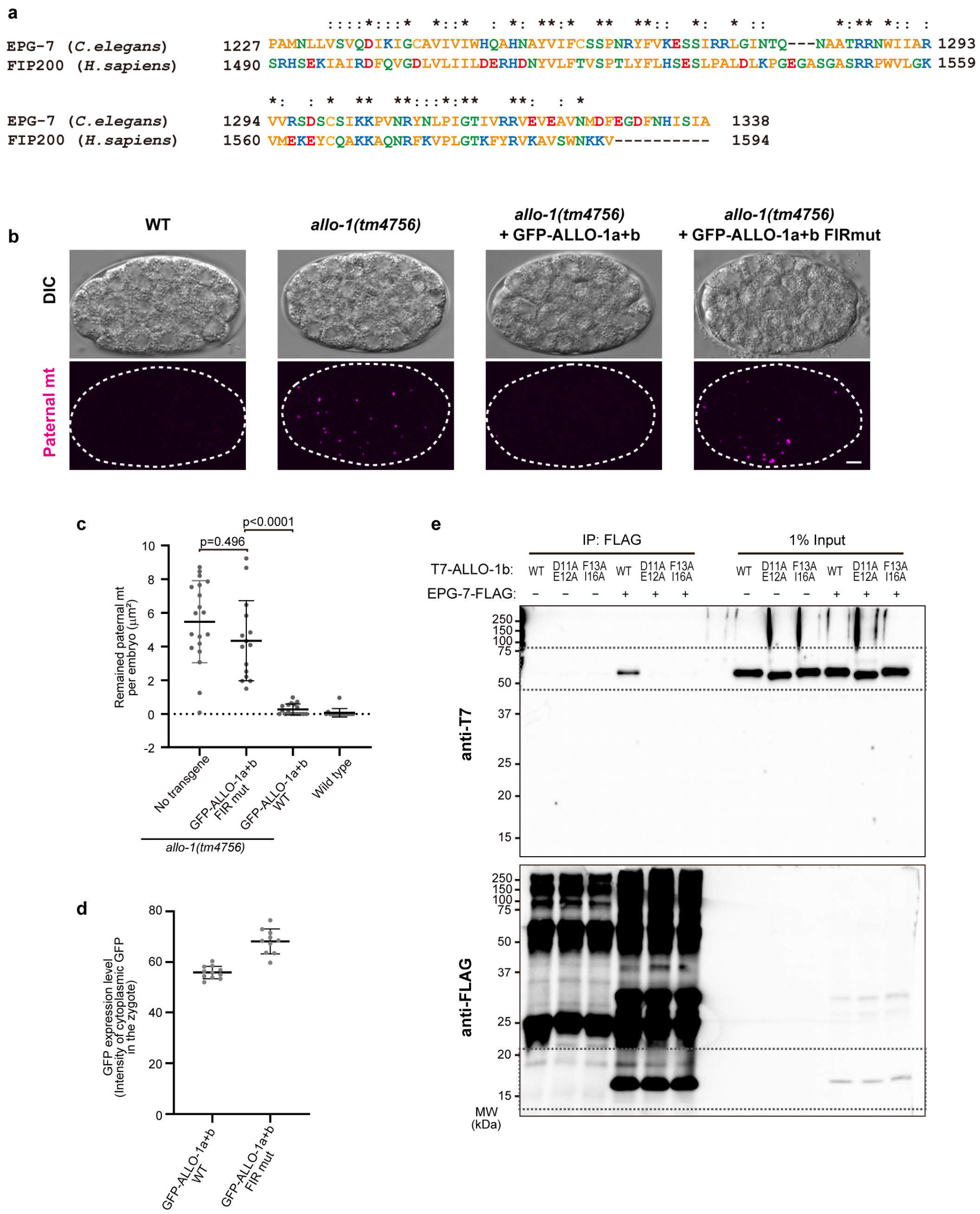

Supplementary Fig. 7. FIP200-interacting region (FIR) motif of ALLO-1 is necessary for degradation of paternal mitochondria

a) Sequence alignment of Claw domains derived from FIP200 and EPG-7. Asterisks and colons indicate conserved and similar residues, respectively. **b, c)** FIR motif of ALLO-1 is necessary for the clearance of paternal mitochondria. The 32–64-cell stage embryos were observed (b), and the remaining paternal mitochondria (magenta) were quantified (c). Egg shells are indicated by white dotted lines. To define the embryonic stage, differential interference contrast (DIC) images are also shown. In c, *n* = 19 (no transgene), *n* = 15 (GFP-ALLO-1a+b D11AE12A; FIR mut), *n* = 17 (GFP-ALLO-1a+b), and *n* = 15 (wild type) embryos were analyzed. Scale bar, 5 μm. **d)** Expression level of GFP-ALLO-1. Intensity of cytoplasmic GFP was quantified outside the vicinity of the paternal organelles to compare GFP expression levels between transgenes. *n* = 11 or 10 zygotes for WT or FIRmut, respectively. **e)** Unprocessed immunoblots of in vitro binding assay. Cropped images from Fig. 7e are indicated by boxes. Error bars represent the mean ± standard deviation (SD). *p* values in c were calculated using Kruskal-Wallis with Steel-Dwass pairwise comparison test.

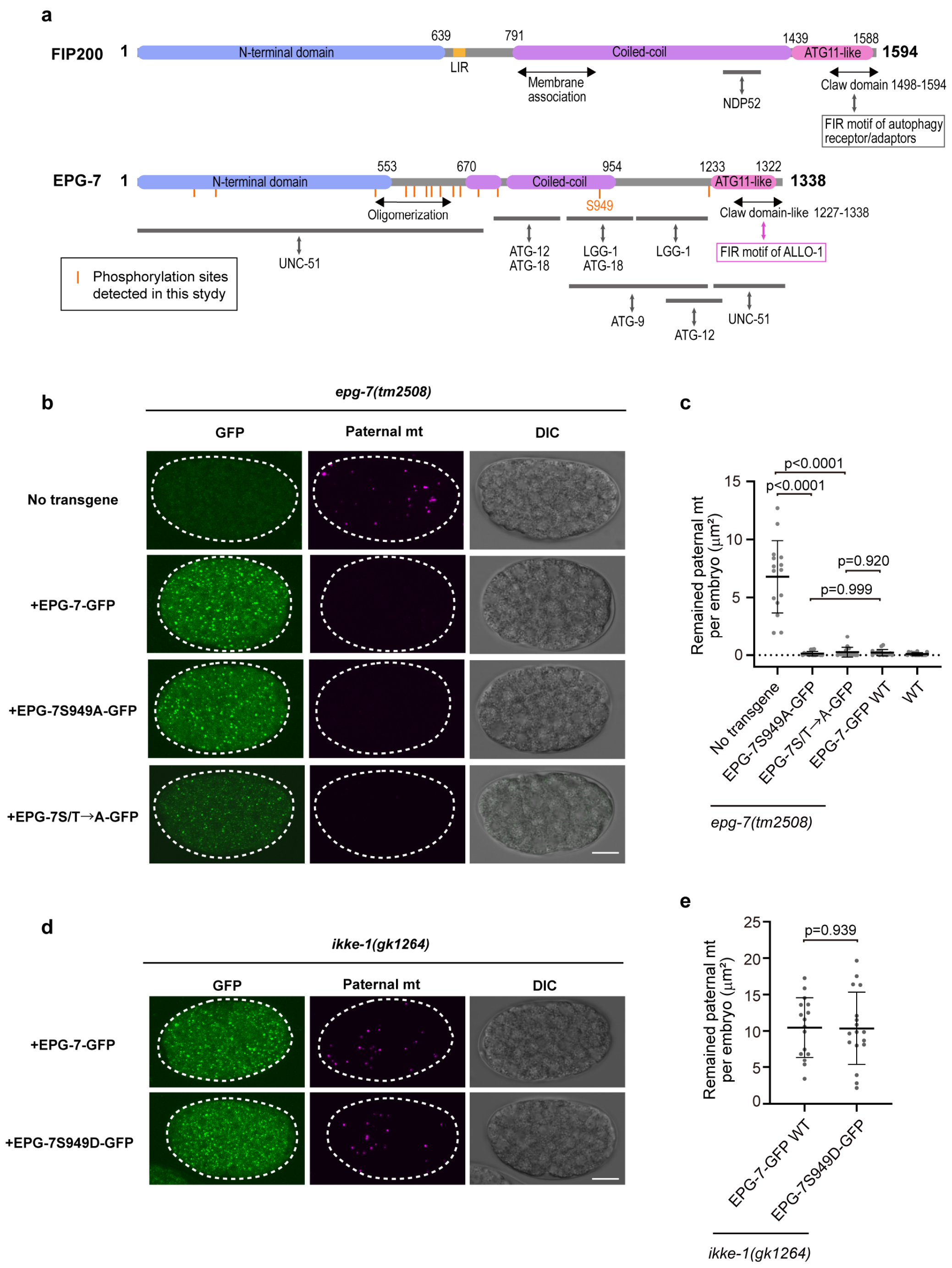

Supplementary Fig. 8. Phosphorylation of EPG-7

a) Structures of mammalian FIP200 and EPG-7. Double-headed arrows in gray indicate the protein-to-protein interaction previously reported. [21, 22, 23, 24, 25, 38] The double-headed arrow in magenta indicates the interaction with ALLO-1 revealed in this study. Phosphorylation sites of EPG-7 detected in this study were indicated by orange bars. **b, c)** Alanine substitution at S949 and all predicted phosphorylation sites in EPG-7 did not affect paternal mitochondrial degradation. The 32–64-cell embryos were dissected from the *epg-7(tm2508)* mutant expressing EPG-7-GFP (wild type, S949A or S/T→A mutants; green), and clearance of paternal mitochondria (HSP-6-mCherry; magenta) were observed. In c, paternal mitochondria remaining in the 32–64-cell stage embryos were quantified as the area showing a fluorescent signal per embryo. n = 15 (no transgene), n = 16 (EPG-7S949A-GFP), n = 18 (EPG-7S/T→A-GFP), n = 15 (EPG-7-GFP WT), and n = 17 (wild-type) embryos. **d, e)** Aspartic acid substitution of S949 in EPG-7 did not rescue

the phenotype of *ikke-1* mutant. The 32–64-cell embryos were dissected from the *ikke-1(gk1264)* mutant expressing EPG-7-GFP (wild type or S949D; green), and clearance of paternal mitochondria (HSP-6-mCherry; magenta) was observed. In e, paternal mitochondria remaining in the 32–64-cell stage embryos were quantified as the area showing a fluorescent signal per embryo. n = 16 (EPG-7-GFP WT) and n = 17 (EPG-7S949D-GFP) embryos. White dotted line indicates the outline of the egg shell. To define the embryonic stage, differential interference contrast (DIC) images are also shown. Error bars represent the mean ± standard deviation (SD). Scale bar, 5 μm. p values were calculated using Kruskal-Wallis with Steel-Dwass pairwise comparison test (c) or two-tailed unpaired t-test (e).

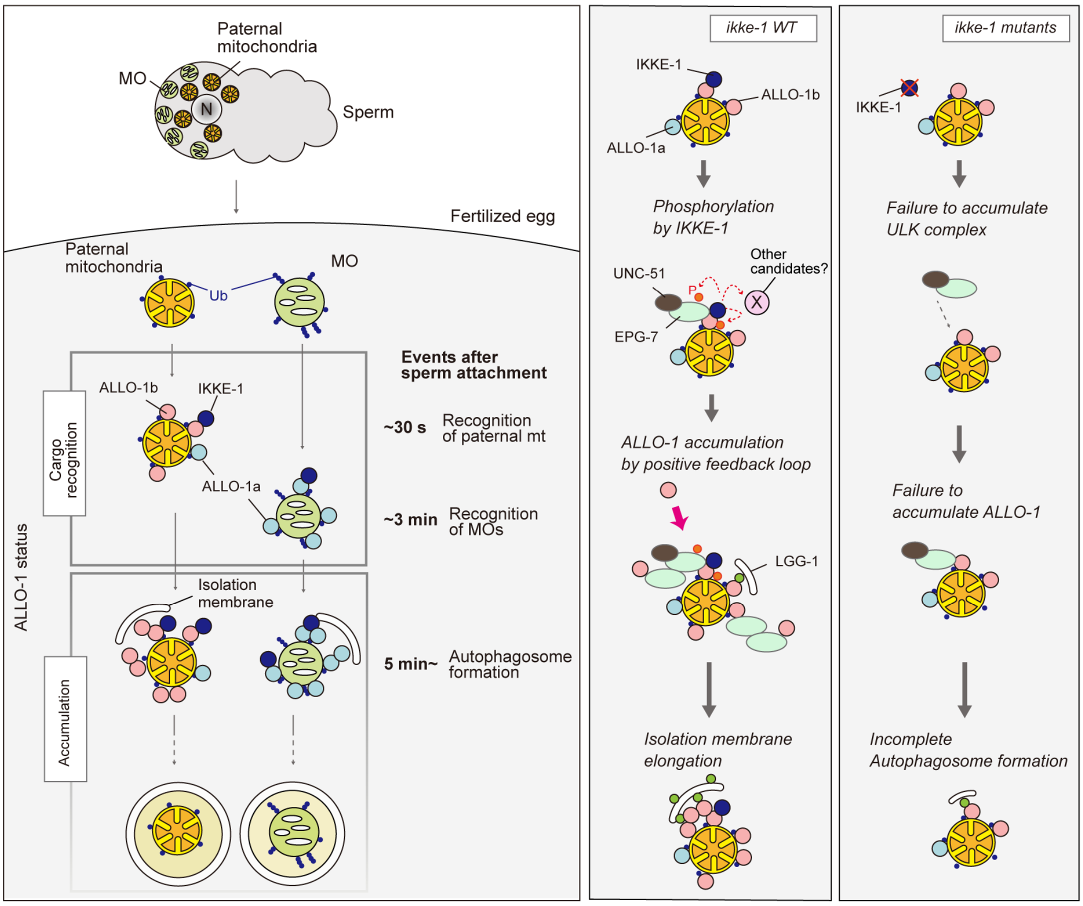

Supplementary Fig. 9. Schematic representation of the mechanism of ALLO-1 localization around paternal organelles during allophagy

Ub; ubiquitin, MO; membranous organelle, P; phosphorylation.

Gene symbol	Mammalian homolog	Annotated Sequence	Modifications	log_2 ratio (*wild-type/ikke-1*)	P-value (-log_{10})
EPG-7	FIP200	[R].EKEQLESLIR.[Q]	2xTMT6plex [N-Term; K2]; 1xPhospho [S7(100)]	3.441793742	7.24295781
EPG-7	FIP200	[K].EQLESLIR.[Q]	1xTMT6plex [N-Term]; 1xPhospho [S5(100)]	2.866614338	3.757847625
EPG-1	ATG13	[R].NHSFPFVNLLQSAYNPANGTK.[K]	2xTMT6plex [N-Term; K21]; 2xDeamidated [Q11(99.3); N18(99.3)]; 1xPhospho [S3(100)]	0.590173927	1.842117878
EPG-1	ATG13	[R].NHSFPFVNLLQSAYNPANGTK.[K]	2xTMT6plex [N-Term; K21]; 1xPhospho [S3(100)]	0.484423562	2.341886518
EPG-1	ATG13	[R].NHSFPFVNLLQSAYNPANGTK.[K]	2xTMT6plex [N-Term; K21]; 1xDeamidated [N18(100)]; 1xPhospho [S3(100)]	0.473296897	1.953136642
UNC-51	ULK1	[K].SATTANIQGIPR.[G]	1xTMT6plex [N-Term]; 1xPhospho [T3(98.8)]	0.458940321	2.727290269
ATG-2	ATG2	[K].TSVSSSEGDIAR.[M]	1xTMT6plex [N-Term]; 1xPhospho [S/T]	0.3272327	1.301713193
EPG-6	WIPI	[R].SRGSSSIVK.[I]	2xTMT6plex [N-Term; K9]; 1xPhospho [S4(99.6)]	0.175571565	1.392006032

Supplementary Table 1. Phosphopeptides of autophagy-related genes with reduced phosphorylation levels in the *ikke-1* mutant

Phosphopeptides ($p < 0.05$) were selected from those detected using tandem mass tag (TMT)-based proteomics. p values were calculated from the abundance using two-tailed Student's t-test.

Gene symbol	Annotated Sequence	Modifications	log_2 ratio (*wild-type/ikke-1*)	P-value (-log_{10})
ALLO-1	[K].SNSNHEIEENK.[K]	2xTMT6plex [N-Term; K11]; 1xPhospho [S3(99.6)]	0.212039875	0.626851434
ALLO-1	[K].MSESTLLK.[S]	2xTMT6plex [N-Term; K8]; 1xPhospho [S4(100)]	0.177524566	0.505058264
ALLO-1	[R].QLGVLEDFSS.[-]	1xTMT6plex [N-Term]; 1xPhospho [S]	-0.164343969	0.343899289
ALLO-1	[R].APSPVGSK.[A]	2xTMT6plex [N-Term; K8]; 1xPhospho [S3(100)]	-0.108225256	0.221093641
ALLO-1	[R].TSLLLVSELK.[S]	2xTMT6plex [N-Term; K10]; 1xPhospho [S2(99.7)]	0.107747352	0.196661159
ALLO-1	[K].APESLKDEDPDRTPEASIVETPLLTETLK.[E]	3xTMT6plex [N-Term; K6; K29]; 1xPhospho [T13(100)]	0.011716303	0.014276571

Supplementary Table 2. Phosphopeptides of ALLO-1 detected in the TMT-based proteomics
Phophopeptides or ALLO-1 were selected from those detected using TMT-based proteomics. *p* values were calculated from the abundance using two-tailed Student's *t*-test.

Positions in EPG-7	Annotated Sequence	Modifications	Digestive Enzymes
EPG-7 [204-231]	[R].RIEVPSEIFVKPASLMTSTSTPINPDEK.[C]	1xPhospho [T/S]	Trypsin
EPG-7 [642-669]	[R].QQSSQDLHHVGSAVSSDTSLLGHETPVK.[M]	1xPhospho [S/T]	Trypsin
EPG-7 [205-231]	[R].IEVPSEIFVKPASLMTSTSTPINPDEK.[C]	1xPhospho [S/T]	Trypsin
EPG-7 [1180-1212]	[K].VSTYQGMEESFYQPMAASTIQVATSPSELEVER.[S]	1xPhospho [S25(98.5)]	Trypsin
EPG-7 [600-633]	[R].SIPYVPSLQQLEGLDGPAPGSSAPISIPNSTSSR.[I]	1xPhospho [S1(100)]	Trypsin
EPG-7 [734-744]	[K].MQNIVANMTPR.[N]	1xPhospho [T9(100)]	Trypsin
EPG-7 [943-952]	[R].EKEQLESLIR.[Q]	1xPhospho [S7(100)]	Trypsin
EPG-7 [204-231]	[R].RIEVPSEIFVKPASLMTSTSTPINPDEK.[C]	1xPhospho [S/T]	Trypsin
EPG-7 [546-556]	[R].VESFLTDEPMR.[M]	1xPhospho [S3(100)]	Trypsin
EPG-7 [560-594]	[R].SHFNYSPAAWLSEDGGDSSPVPQPLMCRSPENALR.[E]	1xCarbamidomethyl [C27]; 2xPhospho [S29(100); S/Y]	Trypsin
EPG-7 [560-587]	[R].SHFNYSPAAWLSEDGGDSSPVPQPLMCR.[S]	1xCarbamidomethyl [C27]; 1xPhospho [S/Y]	Trypsin
EPG-7 [560-594]	[R].SHFNYSPAAWLSEDGGDSSPVPQPLMCRSPENALR.[E]	1xCarbamidomethyl [C27]; 1xPhospho [S29(100)]	Trypsin
EPG-7 [600-633]	[R].SIPYVPSLQQLEGLDGPAPGSSAPISIPNSTSSR.[I]	1xPhospho [S/Y/T]	Trypsin
EPG-7 [546-556]	[R].VESFLTDEPMR.[M]	1xOxidation [M10]; 1xPhospho [S3(99.4)]	Trypsin
EPG-7 [734-744]	[K].MQNIVANMTPR.[N]	1xPhospho [T9(100)]	Trypsin
EPG-7 [560-594]	[R].SHFNYSPAAWLSEDGGDSSPVPQPLMCRSPENALR.[E]	1xCarbamidomethyl [C27]; 3xPhospho [S29(100); S/Y]	Trypsin
EPG-7 [600-633]	[R].SIPYVPSLQQLEGLDGPAPGSSAPISIPNSTSSR.[I]	2xPhospho [S7(97.6); S/Y/T]	Trypsin
EPG-7 [614-646]	[L].DGPAPGSSAPISIPNSTSSRINFKQSGRQQSSQ.[D]	1xPhospho [S/T]	Asp-N
EPG-7 [691-700]	[N].DSNDSIDSLL.[D]	1xPhospho [S8(100)]	Asp-N
EPG-7 [658-671]	[S].DTSLLGHETPVKME.[D]	1xPhospho [T9(100)]	Asp-N
EPG-7 [647-657]	[Q].DLHHVGSAVSS.[D]	1xPhospho [S7(100)]	Asp-N
EPG-7 [585-603]	[L].MCRSPENALRESQCRSIPY.[V]	2xCarbamidomethyl [C2; C14]; 1xPhospho [S/Y]	Chymotrypsin
EPG-7 [570-584]	[W].LSEDGGDSSPVPQPL.[M]	1xPhospho [S9(100)]	Chymotrypsin
EPG-7 [663-675]	[L].GHETPVKMEDTVL.[M]	1xPhospho [T4(100)]	Chymotrypsin
EPG-7 [177-191]	[L].VHSKSEELIEKSDTL.[L]	1xPhospho [S/T]	Chymotrypsin

Supplementary Table 3. Phosphorylation sites of EPG-7 detected by LC-MS/MS

Phosphopeptides of EPG-7 detected using liquid chromatography tandem mass spectrometry (LC-MS/MS).

Mutant strains	Origin
N2 (wild-type)	Caenorhabditis Genetic Center
ikke-1(gk1264)	Caenorhabditis Genetic Center
unc-119(ed3)	Caenorhabditis Genetic Center
him-5 (e1490)	Caenorhabditis Genetic Center
ikke-1(tm4102)	S. Mitani
allo-1(tm4756)	S. Mitani
epg-7(tm2508)	S. Mitani
ikke-1(syb2844)	This study
unc-51-gfp(syb2940)	This study
**Transgenes**	
dkIs851[Ppie-1::gfp::allo-1b, unc-119(+)]	This study
dkIs890[Ppie-1::gfp::allo-1a, unc-119(+)]	This study
dkIs926[Ppie-1::epg-7::gfp, unc-119(+)]	This study
dkIs928[Ppie-1::epg-7 S949A::gfp, unc-119(+)]	This study
dkIs985[Ppie-1::gfp::allo-1 a+b D11AE12A, unc-119(+)]	This study
dkIs1011[Ppie-1::gfp::allo-1b D11AE12A, unc-119(+)]	This study
dkIs1015[Ppie-1::epg-7 S/T→A::gfp]	This study
dkIs1128[Ppie-1::epg-7S949D::gfp, unc-119(+)]	This study
dkIs1122 [Ppie-1::gfp::allo-1a(355-388), unc-119(+)]	This study
dkIs1124 [Ppie-1::gfp::allo-1b(355-402), unc-119(+)]	This study
dkIs698[Pspe-11::hsp-6::mCherry, unc-119(+)]	Sato M. et al., Nat Cell Biol., 20(1), 81-91 (2018)
dkIs737[Ppie-1::gfp::ikke-1, unc-119(+)]	Sato M. et al., Nat Cell Biol., 20(1), 81-91 (2018)
dkIs811[Ppie-1::gfp::allo-1a+b, unc-119(+)]	Sato M. et al., Nat Cell Biol., 20(1), 81-91 (2018)
dkIs836[Ppie-1::gfp::allo-1b C-term, unc-119(+)]	Sato M. et al., Nat Cell Biol., 20(1), 81-91 (2018)
dkIs398[Ppie-1::gfp::lgg-1, unc-119 (+)]	Sato, M. & Sato, K. Science 334, 1141-1144 (2011)
dkIs623[Pspe-11::hsp-6::gfp, unc-119 (+)]	Sato, M. & Sato, K. Science 334, 1141-1144 (2011)

Supplementary Table 4. *Caenorhabditis elegans* strain list used in this study

Oligonucleotide	Source
**Forward primer for cloning allo-1 common region (1-354) or allo-1 N-term;** GGGGACAACTTTGTACAAAAAAGTTGTGAACGATCCGCTTGACTCCC	Sato et al., 2018
**Reverse primer for cloning allo-1 common region (1-354);** AAGTTGAGATTTCAGCTCGC	This paper
**Forward primer for cloning allo-1a specific region (355-388) or allo-1b specific region (355-402);** CACTTCTTCTTGTGAGCGAG	This paper
**Reverse primer for cloning allo-1a specific region (355-388);** GGGGACAACTTTGTACAAGAAAGTTGTTAGCTCGAGAAGTCTTCCA	This paper
**Reverse primer for cloning allo-1b specific region (355-402) or allo-1b C-term;** GGGGACAACTTTGTACAAGAAAGTTGTTATTGGTGGGAGATAAAAT	Sato et al., 2018
**Forward primer for cloning allo-1b C-term;** GGGGACAACTTTGTACAAAAAAGTTGTGAAGAAACTATCTGAGAAAA	Sato et al., 2018
**Reverse primer for cloning allo-1 N-term;** **GGGGACAACTTTGTACAAGAAAGTTGTTAGTTTTCTTCAATTTCATGG**	Sato et al., 2018
**Forward primer for cloning epg-7-gfp and epg-7S/T→A-gfp (epg-7 moiety);** caaattttcttttccagatgATAATGTATCATATATTCTAC	This paper
**Reverse primer for cloning epg-7-gfp and epg-7S/T→A-gfp (epg-7 moiety);** agttcttctcctttactcattgcagcGGCAATAGAAATATGATTGAAG	This paper
**Forward primer for cloning epg-7-gfp (gfp moiety);** ATGAGTAAAGGAGAAGAACT	This paper
**Reverse primer for cloning epg-7-gfp (gfp moiety);** tggaaaatacggcaaaattaTTTGTATAGTTCATCCATGC	This paper
**Forward primer for cloning epg-7 C-term (1227-1338);** GGGGACAACTTTGTACAAAAAAGTTGctCCCGCCATGAATCTTTTGGT	This paper
**Reverse primer for cloning epg-7 C-term (1227-1338);** GGGGACAACTTTGTACAAGAAAGTTGTCAGGCAATAGAAATATGATTG	This paper
**Forward primer for site-direct mutagenesis of allo-1b N10A;** CTCTCGGCTGACGAATTCGAGATAATCG	This paper
**Reverse primer for site-direct mutagenesis of allo-1b N10A;** GGAGTCAAGCGGATCGTT	This paper
**Forward primer for site-direct mutagenesis of allo-1b D11A;** CTCTCGAATGCCGAATTCGAGATAATCG	This paper
**Reverse primer for site-direct mutagenesis of allo-1b D11A;** GGAGTCAAGCGGATCGTT	This paper
**Forward primer for site-direct mutagenesis of allo-1b E12A;** CTCTCGAATGACGCATTCGAGATAATCG	This paper
**Reverse primer for site-direct mutagenesis of allo-1b E12A;** GGAGTCAAGCGGATCGTT	This paper
**Forward primer for site-direct mutagenesis of allo-1b D11A E12A;** CTCTCGAATGCCGCATTCGAGATAATCG	This paper
**Reverse primer for site-direct mutagenesis of allo-1b D11A E12A;** GGAGTCAAGCGGATCGTT	This paper
**Forward primer for site-direct mutagenesis of allo-1b F13A I16A;** CTCTCGAATGACGAAGCCGAGATAGCCGAGACTTTTGACC	This paper
**Reverse primer for site-direct mutagenesis of allo-1b F13A I16A;** GGTCAAAAGTCTCGGCTATCTCGGCTTCGTCATTCGAGAG	This paper
**Forward primer for site-direct mutagenesis of epg-7 S949A;** GCGTTGATCCGCCAGATGCCAGAAG	This paper
**Reverse primer for site-direct mutagenesis of epg-7 S949A;** CTCCAGCTGCTCCTTCTCACGAT	This paper
**Forward primer for site-direct mutagenesis of epg-7 S949D;** GATTTGATCCGCCAGATGCCAG	This paper
**Reverse primer for site-direct mutagenesis of epg-7 S949D;** CTCCAGCTGCTCCTTCTCAC	This paper
**Forward primer of 1st PCR of allo-1b or its mutation forms (D11AE12A and F13A I16A) for creating the template of Cell-Free Protein Synthesis Kit;** CCAGCAGGGAGGTACTATGAACGATCCGCTTGACTC	This paper
**Reverse primer of 1st PCR of allo-1b or its mutation forms (D11AE12A and F13A I16A) for creating the template of Cell-Free Protein Synthesis Kit;** GGCCCGCCCGAGCACTTATTCTATTATTGGTGGGAGATAAAATGAAC	This paper
**Forward primer of 2nd PCR of allo-1b or its mutation forms (D11AE12A and F13A I16A) for creating the template of Cell-Free Protein Synthesis Kit (2nd_NF2);** CCCGCGAAATTAATACGACTCACTATAG	NUProtein
**Forward primer of 2nd PCR of allo-1b or its mutation forms (D11AE12A and F13A I16A) for creating the template of Cell-Free Protein Synthesis Kit (2nd_T7_NF1);** CGACTCACTATAGGGCTCACCTATCTCTCTACACAAAACATTTCCCTACATACAACTTTCAACTTCCTATTATGGC TAGCATGACTGGTGGACAGCAAATGGGTCTCCAGCAGGGAGGTACTATG	This paper
**Reverse primer of 2nd PCR of allo-1b or its mutation forms (D11AE12A and F13A I16A) for creating the template of Cell-Free Protein Synthesis Kit (2nd_NCR_U01);** TTTTTTTTTTTTTTTTGGCCCGCCCGAGCAC	NUProtein
**Forward primer for cloning allo-1a(355-388);** AGTTGTGGACCAACAGGAGAGCACCC	This paper
**Reverse primer for cloning allo-1a(355-388);** TGTTGGTCCACAACTTTTTTGTACAAAGTTGGCA	This paper
**Forward primer for cloning allo-1b(355-402);** AAAGTTGTGCTCGCAGGACCAACAGGAG	This paper
**Reverse primer for cloning allo-1b(355-402);** TGCGAGCACAACTTTTTTGTACAAAGTTGGCA	This paper

Supplementary Table 5. Oligonucleotides used in this study
