# Supplementary material for: ALLO-1- and IKKE-1-dependent positive feedback mechanism promotes the initiation of paternal mitochondrial autophagy
Source: Nat Commun. 2024 Feb 17;15:1460. doi: 10.1038/s41467-024-45863-2 (PMC10874384; doi:10.1038/s41467-024-45863-2)
Supplement: Supplementary file 3 — Description of Additional Supplementary Files [file 41467_2024_45863_MOESM3_ESM.pdf]

Description of Additional Supplementary Files

File Name: Supplementary Data 1
Description: Proteins detected using tandem mass tag (TMT)-based proteomics.

File Name: Supplementary Data 2
Description: Phosphopeptides of EPG-7 detected using liquid chromatography tandem mass spectrometry (LC-MS/MS).

File Name: Supplementary Movie 1
Description: Time-lapse imaging movies of allophagy factors shown in Fig. 3.
Time-lapse imaging of GFP-tagged allophagy factors (green) and mCherry-tagged sperm-derived mitochondria (HSP-6-mCherry; magenta). Images were obtained every 15 s (for GFP-ALLO-1a, GFP-ALLO-1b, GFP-IKKE-1, and EPG-7-GFP) or 20 s (for GFP-LGG-1), and the movie was played at 10 frames per second (fps). Scale bars, 10 µm.

File Name: Supplementary Movie 2
Description: Time-lapse imaging movies of GFP-ALLO-b shown in Fig. 4c.
Time-lapse imaging of GFP-tagged ALLO-1b and mCherry-tagged sperm-derived mitochondria (HSP-6-mCherry; magenta) in the *ikke-1* mutant. Images were obtained every 15 s and the movie was played at 10 frames per second (fps). Scale bars, 10 µm.

File Name: Supplementary Movie 3
Description: 3D reconstruction of super-resolution images shown in Fig. 5f.
3D projection and 3D reconstruction images were created by Fiji software. Scale bars, 2 µm.
