# Supplementary material for: ALLO-1- and IKKE-1-dependent positive feedback mechanism promotes the initiation of paternal mitochondrial autophagy
Source: Nat Commun. 2024 Feb 17;15:1460. doi: 10.1038/s41467-024-45863-2 (PMC10874384; doi:10.1038/s41467-024-45863-2)
Supplement: Supplementary file 9 — Reporting Summary [file 41467_2024_45863_MOESM9_ESM.pdf]

nature portfolio

Corresponding author(s): Miyuki Sato and Ken Sato

Last updated by author(s): Feb 1, 2024

Reporting Summary

Nature Portfolio wishes to improve the reproducibility of the work that we publish. This form provides structure for consistency and transparency in reporting. For further information on Nature Portfolio policies, see our Editorial Policies and the Editorial Policy Checklist.

Statistics

For all statistical analyses, confirm that the following items are present in the figure legend, table legend, main text, or Methods section.

n/a	Confirmed	
☐	☒	The exact sample size (*n*) for each experimental group/condition, given as a discrete number and unit of measurement
☐	☒	A statement on whether measurements were taken from distinct samples or whether the same sample was measured repeatedly
☐	☒	The statistical test(s) used AND whether they are one- or two-sided<br>*Only common tests should be described solely by name; describe more complex techniques in the Methods section.*
☒	☐	A description of all covariates tested
☒	☐	A description of any assumptions or corrections, such as tests of normality and adjustment for multiple comparisons
☐	☒	A full description of the statistical parameters including central tendency (e.g. means) or other basic estimates (e.g. regression coefficient) AND variation (e.g. standard deviation) or associated estimates of uncertainty (e.g. confidence intervals)
☐	☒	For null hypothesis testing, the test statistic (e.g. *F*, *t*, *r*) with confidence intervals, effect sizes, degrees of freedom and *P* value noted<br>*Give P values as exact values whenever suitable.*
☒	☐	For Bayesian analysis, information on the choice of priors and Markov chain Monte Carlo settings
☒	☐	For hierarchical and complex designs, identification of the appropriate level for tests and full reporting of outcomes
☒	☐	Estimates of effect sizes (e.g. Cohen's *d*, Pearson's *r*), indicating how they were calculated

Our web collection on statistics for biologists contains articles on many of the points above.

Software and code

Policy information about availability of computer code

Data collection	1. Confocal images: FV10-ASW (v4.2), 2. Time-lapse imaging: Micro-Manager2.0gamma (v10.1.1), iQ (Andor), 3. Immunoblotting: Fusion Solo 7S, 4. Super-resolution images: FV31S-SW (v2.6.1.243), 5. Mass analysis: Xcalibur 4.3
Data analysis	Graphpad Prism 8 (v8.4.3), EasyR (v1.63), Fiji (ImageJ v1.54f), FV10-ASW (v4.2), FV31S-SW (v2.6.1.243), Proteome Discoverer (v2.1, v2.4)

For manuscripts utilizing custom algorithms or software that are central to the research but not yet described in published literature, software must be made available to editors and reviewers. We strongly encourage code deposition in a community repository (e.g. GitHub). See the Nature Portfolio guidelines for submitting code & software for further information.

Data

Policy information about availability of data

All manuscripts must include a data availability statement. This statement should provide the following information, where applicable:

- Accession codes, unique identifiers, or web links for publicly available datasets
- A description of any restrictions on data availability
- For clinical datasets or third party data, please ensure that the statement adheres to our policy

The MS proteomics data have been deposited to the ProteomeXchange consortium via the jPOST partner repository with the dataset identifiers PXD048126 (Fig. 7f and g) and PXD048129 (Supplementary Table 3). Source data for graphs (Figs. 2b, 2d, 4b, 5b, 5e, 6b, 6e, 8b, 8d and 8e) and blots (Fig. 7e) are provided with this paper as a Source Data file and Supplementary Fig. 7e, respectively. The imaging data supporting the finding of this study are available from the authors upon reasonable request.

Research involving human participants, their data, or biological material

Policy information about studies with human participants or human data. See also policy information about sex, gender (identity/presentation), and sexual orientation and race, ethnicity and racism.

Reporting on sex and gender	Not involving human participants on this study.
Reporting on race, ethnicity, or other socially relevant groupings	Not involving human participants on this study.
Population characteristics	Not involving human participants on this study.
Recruitment	Not involving human participants on this study.
Ethics oversight	Not involving human participants on this study.

Note that full information on the approval of the study protocol must also be provided in the manuscript.

Field-specific reporting

Please select the one below that is the best fit for your research. If you are not sure, read the appropriate sections before making your selection.

- [x] Life sciences
- [ ] Behavioural & social sciences
- [ ] Ecological, evolutionary & environmental sciences

For a reference copy of the document with all sections, see nature.com/documents/nr-reporting-summary-flat.pdf

Life sciences study design

All studies must disclose on these points even when the disclosure is negative.

Sample size	No sample size determination was performed in this study.
Data exclusions	No data was excluded.
Replication	All experiments were repeated at least twice and all attempts at replication were successful. The number of experiments was described in the Figure legends.
Randomization	No randomization was used.
Blinding	Investigators were not blinded.

Reporting for specific materials, systems and methods

We require information from authors about some types of materials, experimental systems and methods used in many studies. Here, indicate whether each material, system or method listed is relevant to your study. If you are not sure if a list item applies to your research, read the appropriate section before selecting a response.

Materials & experimental systems

n/a	Involved in the study
☐	☒ Antibodies
☒	☐ Eukaryotic cell lines
☒	☐ Palaeontology and archaeology
☐	☒ Animals and other organisms
☒	☐ Clinical data
☒	☐ Dual use research of concern
☒	☐ Plants

Methods

n/a	Involved in the study
☒	☐ ChIP-seq
☒	☐ Flow cytometry
☒	☐ MRI-based neuroimaging

Antibodies

Antibodies used

The following primary antibodies were used for immunoblotting: 1:5,000 Actin [C4] (mouse; Merck-Millipore, MAB1501), 1:2,000 GFP (goat; Fitzgerald, 70R-GG001), 1:10,000 ALLO-1a (rabbit; this study), 1:1,000 ALLO-1b (rabbit; this study), 1:5,000 T7 (rabbit;, MBL, PM022), 1:5,000 FLAG (mouse; Fujifilm Wako Chemicals, 018-22381)
The following secondary antibodies were used for immunoblotting: Peroxidase AffiniPure Goat Anti-Rabbit IgG (H+L) (Jackson Immuno Research Laboratories, 111-035-003), Peroxidase AffiniPure Goat Anti-Mouse IgG (H+L) (Jackson Immuno Research Laboratories, 115-035-003), Donkey anti-Goat IgG HRP conjugate (AP180P, Merck-Millipore)
The following primary antibodies were used for immunostaining: 1:500 ALLO-1a (rabbit; this study), 1:100 ALLO-1b (rabbit; this study), 1:250 anti-MO (mouse; 1CB4,a gift from Dr. S. L'Hernault), 1:100 mCherry (rat; Thermo Fisher Scientific, clone 16D7, M11217), 1:400 GFP (rabbit; MBL, 598).
The following secondary antibodies were used for immunostaining:Goat anti-Rabbit IgG (H+L) Highly Cross-Adsorbed Secondary Antibody, Alexa Fluor Plus 488 (A-32731), Goat anti-Mouse IgG (H+L) Highly Cross-Adsorbed Secondary Antibody, Alexa Fluor 488 (A-11029), Goat anti-Rabbit IgG (H+L) Highly Cross-Adsorbed Secondary Antibody, Alexa Fluor 555 (A-21429), Goat anti-Mouse IgG (H+L) Highly Cross-Adsorbed Secondary Antibody, Alexa Fluor 555 (A-21424), Goat anti-Rat IgG (H+L) Cross-Adsorbed Secondary Antibody, Alexa Fluor 594 (A-11007), Goat anti-Mouse IgG (H+L) Highly Cross-Adsorbed Secondary Antibody, Alexa Fluor Plus 647 (A32728) (all antibodies are from Life Technologies).

Validation

The anti-ALLO-1a and anti-ALLO-1b were validated by immunoblotting and immunostaing (this study). A mouse monoclonal anti-MO antibody 1CB4, was obtained from Dr. S. L'Hernault, who checked its reactivity against membranous organelles (MOs) in C.elegans (Okamoto and Thomson, 1985). Commercially available antibodies were validated for their applications respectively.

Animals and other research organisms

Policy information about studies involving animals; ARRIVE guidelines recommended for reporting animal research, and Sex and Gender in Research

Laboratory animals

Organism: Caenorhabditis elegans.
All strains we used in this study are listed in Supplementary Table 4.
Stage: Whole life (from zygote, embryo, L1, L2, L3, L4, and adult).
We basically used young adult hermaphrodites to observe fertilization. Zygotes and embryos were dissected from young adult hermaphrodites .

Wild animals

No wild animal was included.

Reporting on sex

Not biased in sex.

Field-collected samples

No field-collected sample was included.

Ethics oversight

No ethics oversight is needed in C. elegans study.

Note that full information on the approval of the study protocol must also be provided in the manuscript.

Plants

Seed stocks	No plant was used.
Novel plant genotypes	No plant was used.
Authentication	No plant was used.
